# Kyste géant para-urétral feminine

**DOI:** 10.11604/pamj.2014.19.17.4299

**Published:** 2014-09-08

**Authors:** Amadou Kassogué, Mamadou Coulibaly, Zanafon Ouattara, Alkadri Diarra, Aly Tembely, Kalilou Ouattara, My Hassan Farih

**Affiliations:** 1 Département de Chirurgie, Service d'Urologie, CHU Hassan II, Fès, Maroc; 2 Département de Chirurgie, Service d'Urologie, CHU Gabriel Touré, Bamako, Mali; 3 Département de Chirurgie, Service d'Urologie, CHU Point G, Bamako, Mali

**Keywords:** Kyste géant, para urétral, féminin, chirurgie, giant cyst, Para-urethral, female, surgery

## Abstract

Le kyste géant para-urétral féminin infecté est rarement rapporté dans la littérature. Ce kyste est différent du diverticule sous urétral sur le plan clinique, diagnostique et thérapeutique. Sa pathogénie se confond avec celle des diverticules sous urétraux. Son traitement n'est pas bien codifié, vu sa rareté. Nous rapportons un cas atypique de kyste géant para urétral infecté chez une jeune femme de 26 ans. Le kyste était symptomatique et la patiente a eu un traitement chirurgical. Nous discutons les aspects cliniques, diagnostiques et thérapeutiques de cette entité rare à travers une revue de la littérature.

## Introduction

Le kyste géant para urétral est rare. Il est différent du diverticule sous urétral. Le kyste para urétral est considérablement moins fréquente. En raison de leur caractère le plus souvent asymptomatique, il est rarement diagnostiqué et traité. Cependant, la présence de ce kyste para urétral peut provoquer des signes uro-génitales. Les kystes symptomatiques sont une indication de traitement chirurgical [1]. Nous rapportons un cas atypique de kyste géant para urétral infecté chez une jeune femme de 26 ans et discutons les aspects cliniques, diagnostiques et thérapeutiques de cette entité rare à travers une revue de la littérature.

## Patient et observation

**Figure 1:**
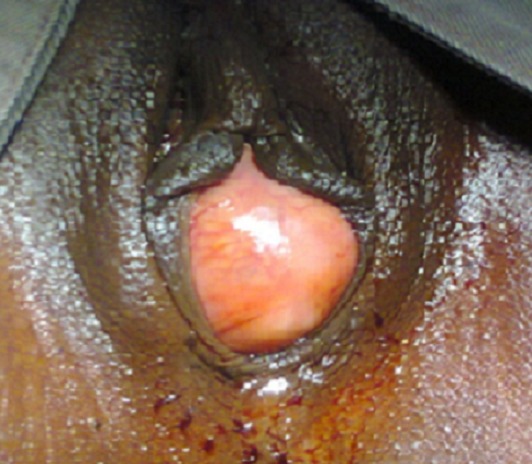
Kyste géant para urétral affleurant à la vulve, le méat urétral est non visible

Patiente de 26 ans, admise en consultation pour tuméfaction endovaginale. Le début de cette tuméfaction endovaginale remontait à trois ans. Elle se manifestait par une symptomatologie urinaire faite de ‘'déviation de jet urinaire’’ et de pollakiurie, sans issue intermittente de pus fétide par le méat urétral et sans autres signes urinaires associés. La patiente dans les antécédents avait signalé une infection génitale à répétition traitée qui était faite de leucorrhée fétide. L'examen clinique retrouvait un périnée propre, une tuméfaction endovaginale qui affleurait la vulve à l'inspection (Figure 1). Cette tuméfaction était rénitente et indolore à la palpation. Le méat urétral n’était pas visible à l'inspection, ce méat urétral n’était visible qu'après le refoulement de la tuméfaction à droite. Le reste de l'examen somatique était sans particularité. A l'expression de la masse il n'y avait pas d'issue de pus à travers le méat urétral. L'examen cytobactériologique des urines (ECBU) avait révélé une infection urinaire à Escherichia coli, sensible à la ciprofloxacine.

**Figure 2:**
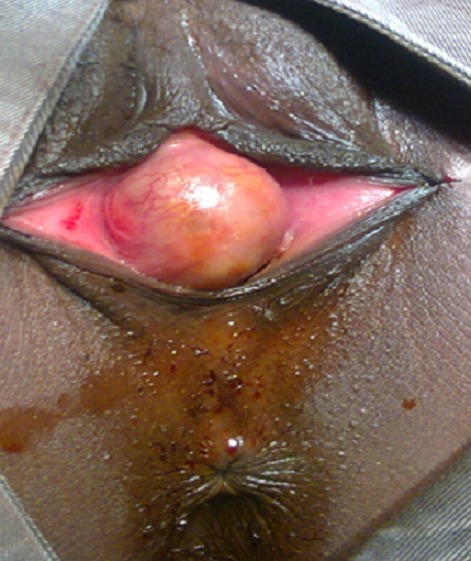
Kyste géant para urétral affleurant la vulve, le méat urétral est non visible; fixation des petites lèvres

**Figure 3:**
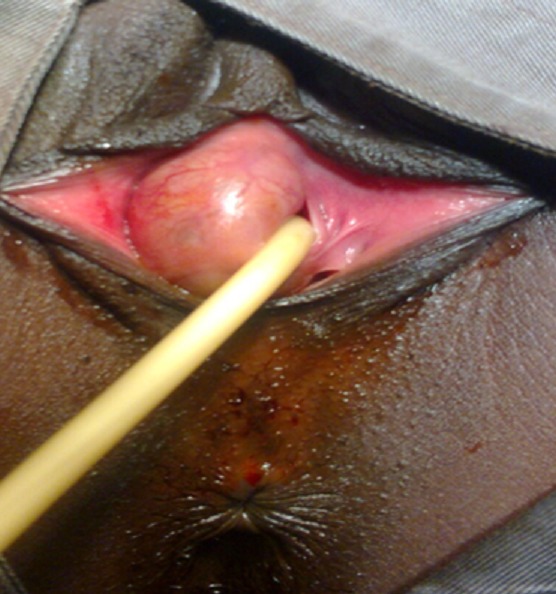
Kyste géant para urétral affleurant la vulve, le méat urétral est non visible spontanément. Sondage vésical. A noter la latéralisation du méat urétral à gauche

**Figure 4:**
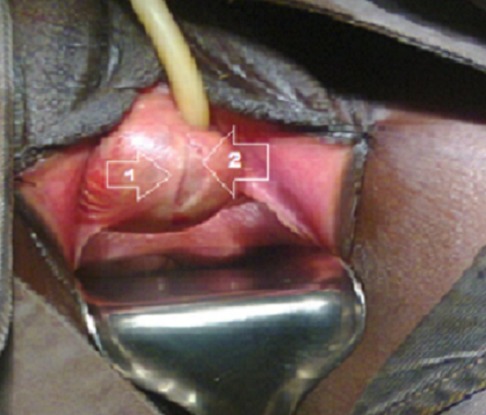
Incision, identification de la paroi vaginale et de la paroi du kyste. 1) Paroi vaginale; 2) Paroi du kyste

**Figure 5:**
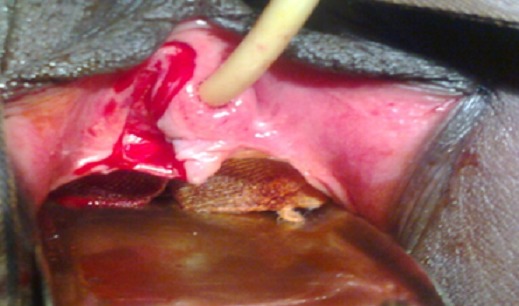
Résection de la paroi vaginale plus kystectomie

**Figure 6:**
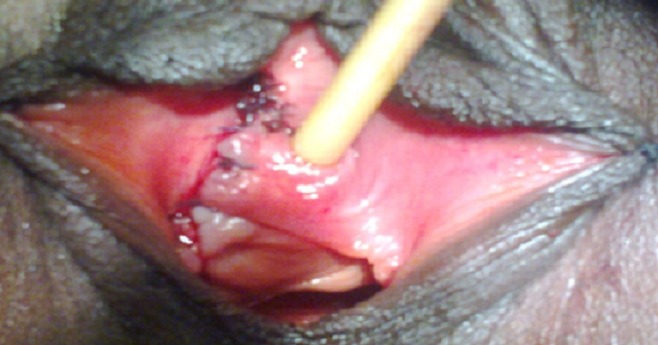
Fin de l'intervention

La patiente a été mise sous ciprofloxacine pendant10 jours (500mgx2/j) avant l'intervention. L’échographie pelvienne et endovaginale avaient montré un kyste à contenu échogène, l'utérus et la vessie étaient sans particularité. Nous n'avons pas réalisé une uretrocystographie (UCG). Le premier temps opératoire consistait à une fixation des petites lèvres (Figure 2), suivi de sondage vésical (Figure 3), puis la réalisation d'une incision vaginale latérale droite (Figure 4). L'exploration per-opératoire a montré: un kyste à contenu purulent ramenant environ 20 cc de pus. On notait l'absence de communication du kyste tant avec l'urètre qu'avec le vagin. Le siège du kyste était à la partie distale et latérale droit de l'urètre. Après nettoyage du contenu de la paroi du kyste avec du sérum salé et de la Bétadine, nous avons procédé à une résection de la paroi du kyste et du vagin (Figure 5) suivi de sa fermeture (Figure 6). Nous avons laissé un tampon vaginal qui a été enlevé à j1. Le drainage des urines était assuré par une sonde urétrale laissée en place pendant 24 heures. La durée d'hospitalisation a été de 48 heures. Les suites opératoires immédiates ont été simples.

## Discussion

Un kyste géant para urétral infecté est rarement rapporté dans la littérature. Ce kyste est différent du diverticule ou de poche sous urétrale sur les plans clinique, diagnostique et thérapeutique. Les pathogénies peuvent être les mêmes mais non documentées dans la littérature actuelle compte tenu de sa rareté. La pathogénie des diverticules urétraux féminins reste discutée. Plusieurs hypothèses ont été avancées, congénitale, iatrogène, traumatique et infectieuse [2]. Les facteurs incriminés dans la genèse des diverticules acquis sont: - l'infection, l'abcédation et la fistulisation des glandes périurétrales dans la lumière urétrale; - les traumatismes obstétricaux et urétraux iatrogènes; - l'obstruction de l'urètre distal [3].

L′hypothèse la plus communément admise pour les diverticules est celle de ROUTH, selon laquelle l′infection et l′obstruction répétées des glandes péri urétrales aboutissent à la formation des kystes sous urétraux. Ces derniers, en se rompant déversent leur contenu dans la lumière urétrale laissant ainsi une cavité qui s′épithélialise secondairement pour former un diverticule [4]. Cette hypothèse est la plus probable chez notre patient sauf que le contenu n’était pas rompu dans la lumière urétrale avec présence d'une cavité d′épithélialisation secondairement pour former un kyste géant para urétral. D'où la présence d'un vrai kyste sans communication avec l'urètre ni le vagin. Ce type de kyste para urétral géant sans communication avec l'urètre est rarissime.

Selon les critères morphologiques proposés par certain auteur [1], les kystes para urétraux peuvent être divisés en quatre groupes, caractérisés par des étiologies différentes: kyste de Muller, kyste du canal de Gartner, kyste de la glande de Skene, kyste acquis de la squamation épithéliale. La différenciation clinique entre ces différents types de kystes est difficile. Les signes observés en cas de kyste para urétral dans la littérature sont: sensation de masse, dyspareunie, dysurie, douleurs périodiques dans la région des organes génitaux externes [1, 5]. Dans notre cas, la patiente avait la symptomatologie urinaire et génitale. Notre patiente rapportait une notion d'infection gynécologique (leucorrhée fétide) à répétition associée à de troubles mictionnels à type de déviation de jet urinaire et de pollakiurie. L'infection urogénitale est la cause la plus probable.

Le diagnostic était clinique et échographique dans notre cas. Le kyste était géant, palpable, et il n'y avait pas d'issue de pus par le méat urétral à l'expression de la masse. La tuméfaction était rénitente permettant de faire le diagnostic différentiel avec le diverticule sous urétral. L’échographie pelvienne et endovaginale avait montré un kyste avec un contenu échogène sans autre anomalie associée. Vu le résultat de l’échographie et de l'examen clinique notamment l'absence d'issue de pus par le méat nous n'avons pas réalisé une UCG. Il est a noté que quelques cas de kyste para urétral ont été rapporté chez le nourrisson [6]. Les quelques rares cas rapportés dans la littérature proposent le traitement chirurgical pour les kystes para urétraux symptomatiques. Notre patiente était symptomatique et a eu un traitement chirurgical, ce qui est cohérente avec la littérature pour les kystes symptomatiques. Le kyste géant para urétral féminin infecté est rarement rapporté dans la littérature. La présence de ce kyste para urétral peut provoquer des symptomatologies uro-génitales.

## Conclusion

Le kyste géant para urétral est rare, son diagnostic se confond avec les diverticules sous urétraux à l'examen clinique. L'infection urogénitale semble être la cause. Les quelques rares cas rapportés dans la littérature proposent le traitement chirurgical pour les kystes para urétraux symptomatiques.
